# Anxiety after Sympathectomy in patients with primary palmar hyperhidrosis may prolong the duration of compensatory hyperhidrosis

**DOI:** 10.1186/s13019-018-0736-3

**Published:** 2018-06-01

**Authors:** Kai Qian, Yong-Geng Feng, Jing-Hai Zhou, Ru-Wen Wang, Qun-You Tan, Bo Deng

**Affiliations:** Department of Thoracic Surgery, Institute of Surgery Research, Daping Hospital, Army Medical University, Chongqing, 400042 People’s Republic of China

**Keywords:** Anxiety, Palmar hyperhidrosis, Compensatory hyperhidrosis

## Abstract

**Background:**

Compensatory hyperhidrosis (CH) is a frequent side effect after sympathectomy for the treatment of primary palmar hyperhidrosis. We determined the effects of demographic and clinical factors which may increase the duration of CH (DCH).

**Methods:**

One hundred twenty-two patients who had undergone sympathectomies from 2014 to 2016 were retrospectively reviewed. Anxiety was evaluated using the State and Trait Anxiety Inventory score. Follow-up evaluations continued until CH remitted. A Cox proportional hazards model was used to determine the association between DCH and variables.

**Results:**

DCH ranged from 5 to 27 weeks (median, 11.47 weeks). Severe CH (HR = 0.318, 95% CI, 0.136–0.741) and exacerbated anxiety 1 month post-operatively (HR = 0.816, 95% CI, 0.746–0.893) may prolong CH. A positive correlation between post-operative anxiety and DCH was common in patients with moderate or severe CH, and in cases with forearm CH.

**Conclusions:**

Pre- and post-operative anxiety should be evaluated, and anti-anxiety treatment is offered to patients with moderate-to-severe CH to shorten the DCH.

**Electronic supplementary material:**

The online version of this article (10.1186/s13019-018-0736-3) contains supplementary material, which is available to authorized users.

## Background

Primary palmar hyperhidrosis (PH) is a condition marked by excessive perspiration, which can be exacerbated by stress and anxiety [1]. Sympathectomy by video-assisted thoracic surgery (VATS) has been used in PH treatment with acceptable results [2]; however, post-operative compensatory hyperhidrosis (CH) is a frequent side effect (morbidity, 44–86% [3]) that significantly lowers the quality of life [4]. CH can result from aberrant function of the sympathetic nervous system after surgery [5], which destroys the nerve flex arch between the sympathetic nervous system and hypothalamus [6]. A systemic review suggested that, as compared with other levels, a T3 or T3–4 sympathectomy can offer better efficacy and satisfaction rate and result in less post-operative CH [7]. Fortunately, post-operative CH will be gradually and finally obliterated [8]; however, the clinical or demographic factors may prolong the duration of CH (DCH) remain unclear.

CH may lead to severe anxiety [9]. Conversely, we speculate that anxiety may affect DCH following sympathectomy. Therefore, we sought to analyze these clinical and demographic factors, including anxiety, which may prolong the DCH.

## Methods

### Patients

The study protocol was reviewed and approved by the Research Ethics Board in Daping Hospital (Chongqing City, P.R. China) [reference no. 20160809], and informed consent was written and obtained from all patients.

From September 2014 to September 2016, there were 122 patients with severe PH, as defined by United States Skin Association [10, 11], who underwent bilateral multiple-level VATS sympathectomy (Table 1). The exclusion criteria were as follows: (i) patients accepted botulinum toxin treatment and Chinese herb treatment prior to operation; (ii) patients with uncorrected bleeding diatheses (international normalized ratio>1.7 and platelet count < 50,000/dL); (iii) pre-operative acute inflammation (serum C-reactive protein concentration > 10 mg/L; white blood cell count > 10 × 10^9^/L); (iv) patients were diagnosed with secondary hyperhidrosis or other neuromuscular diseases; and (v) patients were pregnant or lactating.

**Table 1:** Clinical and demographic characteristics of patients with and without CH^a^

	Patients without CH	Patients with CH	*P* value
	(*n* = 65)	(n = 57)	
Gender			0.323*
Male	40(61.54)	30(52.6)	
Female	25(38.46)	27(47.4)	
Age[years]			0.440*
Mean	21.65	21.33	
SD	5.001	5.601	
BMI[kg/m^2^]			0.006
Mean	22.86	20.04	
SD	1.47	1.784	
Location of CH^b^			–
Head, face, and neck	–	24(42.1)	
Forearm	–	24(42.1)	
Trunk and perineum	–	5(8.8)	
Calves, feet, and thighs	–	4(7.0)	
Degree of CH^**c**^			–
None	65(100)	0	
Mild	–	30(52.6)	
Moderate	–	16(28.1)	
Severe	–	11(19.3)	
Hresult-1 m			0.830*
Completely dry	40(61.5)	44(77.2)	
Significant improvement	25(38.5)	13(22.8)	
Improvement	0	0	
No change	0	0	
SAI-P			0.014*
Mean	37.22	39.86	
SD	3.319	5.749	
SAI-1 m			0.481*
Mean	37.51	40.19	
SD	3.355	8.037	
TAI-P			0.633*
Mean	39.80	40.54	
SD	1.725	5.558	
TAI-1 m			0.697*
Mean	39.75	40.61	
SD	1.714	5.552	
The method of sympathectomy			0.896*
T2-3	32(49.2)	28(49.1)	
T2–4	14(21.5)	11(19.3)	
T3–4	19(29.2)	18(31.6)	

*Note:* ^a^Kolmogorov–Smirnov test for determination of distribution

*Mann–Whitney U test

^b^According to the rule of nines, i.e., guide for resuscitation of burn patients, the location of CH divides into 4 groups [27]

^**c**^Mild: CH is not noticeable, unless under detailed questioning; Moderate: CH is tolerable, but sometimes interferes with daily activities; Severe: CH is intolerable and always interferes with daily activities

*Abbreviations:* BMI: body mass index; CH: compensatory hyperhidrosis; Hresult-1 m: hand effects in post-operative 1 month; SAI-1 m: State Anxiety Inventory score 1 month post-operatively; SAI-P: Pre-operative State Anxiety Inventory score; TAI-1 m: Trait Anxiety Inventory score 1 month post-operatively; TAI-P: Pre-operative Trait Anxiety Inventory score

**Fig. 1:**
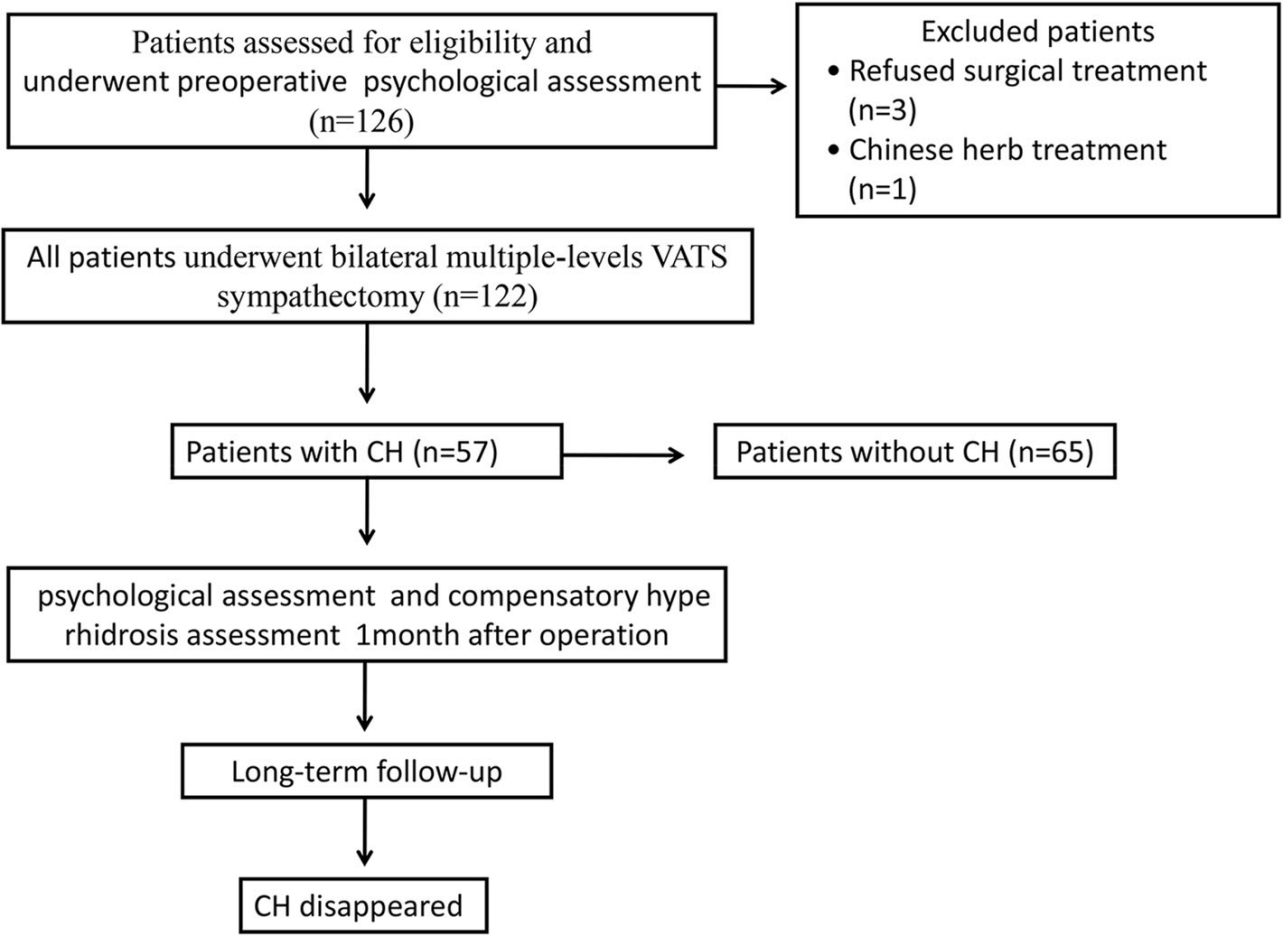
Follow-up strategy of patients following sympathectomy. The psychological assessment included indicators of anxiety, i.e., *SAI-1 m:* State Anxiety Inventory score 1 month post-operatively; *SAI-P:* Pre-operative State Anxiety Inventory score; *TAI-1 m:* Trait Anxiety Inventory score 1 month post-operatively; *TAI-P:* Pre-operative Trait Anxiety Inventory score

The follow-up strategy is shown in Fig. 1. Post-operatively, patients were confirmed to have or not have CH, which was defined as new onset post-operative or worsening sweating in the lower extremities, trunk, axillae, face, and groin [2]. All patients were queried via questionnaire (Hyperhidrosis Disease Severity Scale) [12], as shown in Additional file 1: Table S1. Follow-up was discontinued in patients when CH remitted.

### Bilateral sympathectomy by VATS

**G**eneral anesthesia and a double-lumen endotracheal tube placement was performed. During the operation, patients were placed in the supine position with exposure of both axillary regions. A 1-cm incision was made at the third intercostal space on the anterior axillary line, and a 5-mm diameter thoracoscope (0° telescope; Karl Storz, Germany) was placed via a 12-mm trocar. To identify anatomic landmarks, pneumothoraces were induced in all patients via temporary apnea [13]. An electrocautery hook was inserted to isolate and cut the sympathetic chains at T2–3, T2–4, or T3–4 (Table 1). After sympathectomy, the lung was confirmed to be re-expanded after air was evacuated from the pleural cavity via a 16-Fr catheter [14]. Chest drainage tubes were not placed in all of the patients.

### Anxiety evaluation

Anxiety was evaluated using State-Trait Anxiety Inventory (STAI) [15], differentiating temporary “state-anxiety”(SAI) from long-term quality of “trait-anxiety”(TAI). The STAI can be used in clinical settings to diagnose anxiety and to distinguish anxiety from depressive syndromes. The STAI can be obtained from the publisher (Mind Garden, 855 Oak Grove Avenue, Suite 215, Menlo Park, CA 94025; USA, URL, http://www.mindgarden.com/index.htm). SAI is the temporary and changeable feeling induced by the arousal of the autonomic nervous system (e.g., how a person is feeling at the time of a perceived threat) [16]. TAI is viewed as a predisposition to anxiety, which is a relatively stable personality characteristic [17]. SAI and TAI were measured using a 4-point rating scale for 20 items (1, ‘not at all’; 2, ‘mild’; 3, ‘moderate’; and 4, ‘very’) [18]. Total scores of SAI and TAI varied between 20 and 80.

### Statistical analysis

Kolmogorov–Smirnov test was used for determination of the distribution. The Fisher exact test was used for categorical variables. The t-test or Mann–Whitney U test was used for continuous variables. Univariate and multivariate analyses were performed using a Cox proportional hazards model to determine associations between DCH and potential impact factors. Parameters demonstrating a statistically significant effect on DCH in the univariate Cox model were included for analysis in the multivariate model with forward and stepwise selection. Hazard ratios (HRs) were estimated as relative risk with corresponding 95% confident intervals (CIs). Statistical analyses were performed with SPSS (version 23.0, IBM Corp.: Armonk, NY), with a two-sided *P* < 0.05 considered statistically significant for all reports. The XLSTAT® (Addinsoft company, Pairs, France) was used to assess statistical power.

## Results

### Clinical and demographic characteristics of patients with CH

Among the 122 patients, 57 (46.72%) developed CH after sympathectomies (Table 1). Post-operative follow-up was discontinued until CH remitted, ranging from 5 to 27 weeks (median duration, 11.47 weeks). We chose 1 month post-operatively as the time point to evaluate the psychologic status for the following reasons: (i) anxiety resulting from surgery has resolved; and (ii) CH in some cases resolved within 5 weeks.

The body mass index (BMI) of the CH group were significantly lower, as compared with the non-CH group, even though the BMIs in either group were within the normal range (Table 1). Table 1 shows significant severe pre-operative anxiety status (SAI-p) of the patients with CH as compared with patients without CH (*p* < 0.05).

### Clinical factors prolonged CH

The univariate Cox model indicated that four factors significantly may increase DCH, i.e., CH degree (HR = 0.098; 95% CI, 0.045–0.216), SAI-p (HR = 0.908; 95% CI, 0.854–0.966), SAI-1 m (HR = 0.773; 95% CI, 0.716–0.843), sympathectomy level (HR = 1.698; 95% CI, 1.256–2.295), TAI-p (HR = 0.900; 95% CI, 0.845–0.958), and TAI-1 m (HR = 0.893; 95% CI, 0.838–0.953; Table 2).

**Table 2:** Risk factors and prolonged duration of CH (*n* = 57)

Variables	Univariate analysis		Multivariate analysis	
	HR(95% CI)	*P* value	HR(95% CI)	*P* value
SAI-1 m	0.773(0.716–0.843)	0.000^a^	0.816(0.746–0.893)	0.000^b^
TAI-1 m	0.893(0.838–0.953))	0.001^c^	N.A.	0.874
SAI-P	0.908(0.854–0.966)	0.002^d^	N.A.	0.248
TAI-P	0.900(0.845–0.958)	0.001^e^	N.A.	0.453
Degree	0.098(0.045–0.216)	0.000^f^	0.318(0.136–0.741)	0.002^g^
Location	0.898(0.709–1.138)	0.374	N.A.	N.A.
Sympathectomy level	1.698(1.256–2.295)	0.001^h^	N.A.	0.751

Note: Statistical powers of the Cox model were calculated using XLSTAT (Addinsoft, Inc., New York, NY, USA) and presented as follows: ^a^= 0.965; ^b^= 0.978; ^c^= 0.966; ^d^= 0.939; ^e^= 0.984; ^f^= 0.104; ^g^= 0.624; and ^h^= 0.932. N.A. = not available

A systemic review ^7^ indicated a variety of CH incidences among different sympathectomy levels as follows: T2–3, 57%; T2–4, 38%; and T3–4, 6%. Therefore, we categorized the sympathectomy levels as follows: T2–3 = 1; T2–4 = 2; and T3–4 = 3

The multivariable Cox model also showed that severe CH and a high SAI-1 m score was significantly associated with prolonged CH (HR = 0.318; 95% CI, 0.136–0.741 and HR = 0.816; 95% CI, 0.746–0.893), as shown in Table 2.

### Correlation between SAI-1 m and DCH

**Fig. 2:**
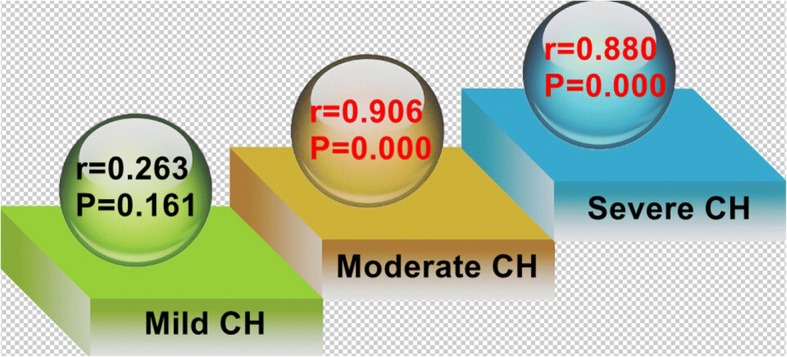
Correlation between SAI-1 m and DCH. SAI-1 m may prolong DCH in the cases with moderate-to-severe CH (*r* = 0.906, *P* = 0.000; and *r* = 0.880, P = 0.000). *Mild:* CH is not noticeable, unless under detailed questioning; *Moderate:* CH is tolerable, but sometimes interferes with my daily activities; *Severe:* CH is intolerable and always interferes with my daily activities

Severe anxiety 1 month post-operatively was significantly associated with prolonged CH in the patients with moderate or severe CH (*r* = 0.906, *P* = 0.000; and *r* = 0.880, P = 0.000); however, there was no correlation in patients with mild CH (Fig. 2).

**Fig. 3:**
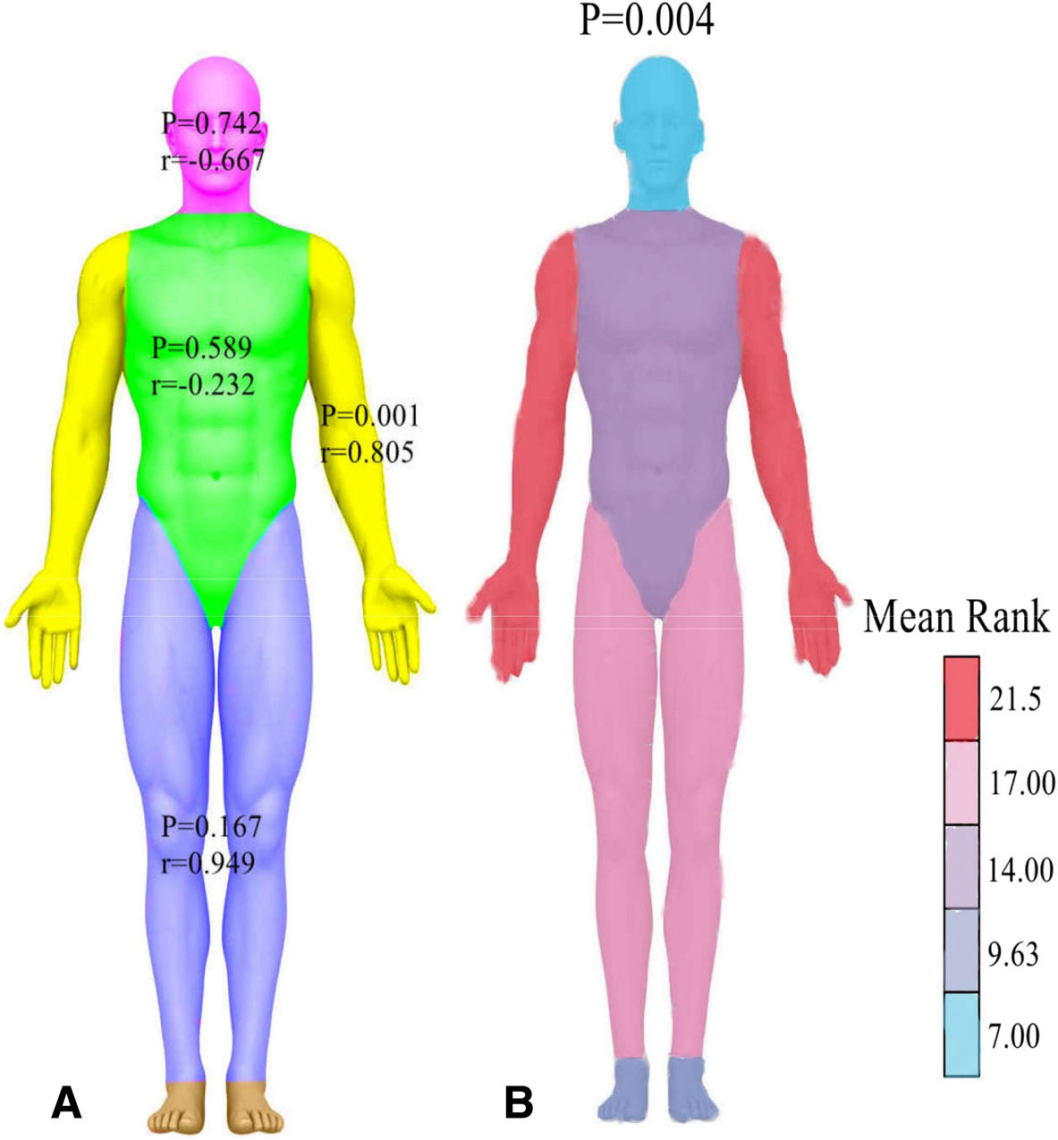
**a** The significant positive correlation (*r* = 0.805; *P* = 0.001) between SAI-1 m and DCH was found in the patients with forearm CH, rather than other anatomic locations; (**b**): Forearm CH was more severe as compared with other anatomic areas (*P* = 0.004)

Severe anxiety 1 month post-operatively was significantly associated with prolonged CH in the patients with forearm CH (*r* = 0.805; *P* = 0.001) (Fig. 3a). Indeed, forearm CH appeared to be more severe compared with other anatomic areas (*P* = 0.004), as compared to Fig. 3b.

## Discussion

Severe CH is one of the refractory complications after sympathectomy for PH treatment, causing patient dissatisfaction and depression [4]. The incidence of CH ranges from 33 to 85%, and CH may persist 6–12 months [19]. In the current study, CH occurred in 57 patients (46.72%) and the medium DCH was 11.47 weeks. To obtain detailed CH information, our follow-up period lasted 52 weeks, indicating a gradual decrease in the intensity of CH.

Sweating is influenced by two factors (emotional stimulation [central control] and environmental temperature [peripheral control]) [12]; however, PH was more likely triggered by emotional stimulation (anxiety and depression), but less related to environmental temperature [20]. Based on the Hospital Anxiety and Depression Scale, Braganca et al. [21] reported that anxiety, rather than depression, is main cause of primary PH. Kumagai et al. [22] showed that less anxiety was accompanied with a lower degree of CH after sympathectomy. Our study revealed more severe pre-operative anxiety in patients with CH compared to patients without CH, and severe post-operative anxiety may be critical to prolong DCH, especially in patients with moderate-to-severe CH.

The incidence of anxiety was reported to be higher in patients with axillary or craniofacial PH compared with other anatomic areas. Nevertheless, we found forearm CH to be more severe compared with other anatomic areas, and exacerbated anxiety resulting from CH may predispose to prolonged CH in these patients.

Before the introduction of sympathectomy, there were a variety of anti-anxiety treatments for PH, such as hypnosis, psychotherapy [23], biofeedback [24], and tranquilizing drugs [24]. Anti-anxiety treatment may reduce negative physiologic manifestations, risk of infection, and the induction of anesthesia, but increase wound healing and promote post-operative recovery [25]. Patients who underwent psychosocial intervention following coronary artery bypass grafting were at reduced risk of adverse post-operative events [26].

In our cohort, there was one patient with insomnia caused by anxiety who was treated with eszopiclone (3 mg/qd) for the 10-day perioperative period. Interestingly, although the patient had severe PH pre-operatively, he did not exhibit CH after sympathectomy. As a result, a randomized clinical trial is warranted to study the impact of pre-operative anti-anxiety treatment on CH and DCH.

In summary, our study has shown that pre- and post-operative anxiety should be evaluated, and anti-anxiety treatment should be administered to patients with moderate-to-severe CH in an effort to shorten the DCH.

## Conclusions

Pre- and post-operative anxiety should be evaluated, and anti-anxiety treatment should be administered to patients with moderate-to-severe CH to shorten the DCH.

## Additional file

Additional file 1: **Table S1.** Post-operative self-assessment questionnaire. (DOCX 13 kb)

## Abbreviations

BMI: Body mass index
CH: Compensatory hyperhidrosis
CI: Confidence interval
HR: Hazard radio
SAI-1 m: State Anxiety Inventory score 1 month post-operatively
SAI-P: Preoperative State Anxiety Inventory score
TAI-1 m: Trait Anxiety Inventory score 1 month post-operatively
TAI-P: Preoperative Trait Anxiety Inventory score
