# Supplementary material for: Anxiety after Sympathectomy in patients with primary palmar hyperhidrosis may prolong the duration of compensatory hyperhidrosis
Source: J Cardiothorac Surg. 2018 Jun 1;13:54. doi: 10.1186/s13019-018-0736-3 (PMC5984730; doi:10.1186/s13019-018-0736-3)
Supplement: Supplementary file 1 — Table S1. Post-operative self-assessment questionnaire. (DOCX 13 kb) [file 13019_2018_736_MOESM1_ESM.docx]

**Postoperative self-assessment questionnaire**

| **Question** | **Response** |
| --- | --- |
| Name |  |
| Age |  |
| Gender | □Male □Female |
| Operation date |  |
| BMI | Height /Weight^2^ ＝ |
| Before operation did you accept other therapy? | □Botulinum toxin treatment  □Drugs  □Chinese herb treatment  □Iontophoresis  □Others |
| **Recurrence of symptoms** |  |
| a. Does the surgery effectively correct your problem with excessive sweating on the palms? | □Yes □No |
| b. If not, how severe is the palmar sweating? | □Completely dry  □Significant improvement  □Improvement  □No change |
| **Compensatory sweating** |  |
| a. Do you have excessive sweating in other areas of your body? | □Yes □No |
| b. If yes, which parts are affected? | □Head[,](http://cn.bing.com/dict/search?q=%2C&FORM=BDVSP6&mkt=zh-cn) face and neck  □Forearm  □Trunk and perineum  □Calves, foot and thighs |
| c. What is its severity(Degree of CH)? | □None  □Mild: CH is not noticeable, unless under detailed questioning;  □Moderate: CH is tolerable but sometimes interferes with daily activities;  □Severe: CH is intolerable and always interferes with daily activities |
